# Mother’s perceptions and concerns over sharing sexual and reproductive health information with their adolescent daughters- A qualitative study among mothers of adolescent girls aged 14–19 years in the developing world, Sri Lanka

**DOI:** 10.1186/s12905-023-02369-1

**Published:** 2023-05-03

**Authors:** D Mataraarachchi, P.K. Buddhika Mahesh, T.E.A. Pathirana, Gayan Ariyadasa, Chamanthi Wijemanne, Indumini Gunatilake, Indika Nupahewa, Ayesha Gunasoma, P.V.S.C. Vithana

**Affiliations:** 1 Family Health Bureau, Ministry of Health, Colombo, Sri Lanka; 2 Provincial Director of Health Services, Mawatha Badulla, Western Province Sri Lanka; 3 Base Hospital, Panadura, Sri Lanka

**Keywords:** Adolescent sexual and reproductive health, Communicating SRH matters, Mothers’ perception

## Abstract

**Introduction:**

Studies across the world have shown that parent-adolescent SRH communication can prevent unhealthy sexual and reproductive health (SRH) practices among adolescents and promote their sexual and reproductive health. Parents have the ability to provide individualized sex education to suit the needs of their children, their families, and societal values. The fact that children have more opportunities in the family, makes parent-based sexuality education of children a better approach to Sri Lankan context.

**Objective:**

To explore mother’s perceptions and concerns over sharing SRH information with their daughters among Sinhalese mothers of adolescent girls aged 14–19 years in Sri Lanka.

**Methodology:**

Six Focus group discussions (FGD) were carried out among mothers of adolescent girls aged 14–19 years. Each focus group discussion included 10–12 participants who were recruited using the purposive sampling method. A focus group discussion guide developed after an extensive literature search and expert opinion was used to retrieve information from mothers. Data management and analysis mainly followed an inductive approach to thematic analysis principles. The findings were presented in narrative form using respondents’ quotes in their own words and were developed into codes and themes.

**Results:**

The mean age of the participants was 43.5 years while 62.4% (n = 40) were educated above the Ordinary level. Analysis of data identified eight main themes from the FGDs. Many mothers thought that sexual and reproductive information is important to adolescent girls. They tried to keep their girl adolescents informed about Adolescent sexual and reproductive health (ASRH) issues. They preferred abstinence-only education over abstinence-plus education. Lack of skills and lack of knowledge on adolescent SRH issues was a major challenge identified by the mothers to communicate SRH matters with their children.

**Conclusions and recommendations:**

Although mothers perceived their role as primary sex educators to their children, they were not confident about their knowledge and skills in discussing SRH matters with children. Implementation of interventions to improve mothers’ attitudes and skills in communicating SRH matters with children is recommended.

**Supplementary Information:**

The online version contains supplementary material available at 10.1186/s12905-023-02369-1.

## Introduction

With the onset of puberty, intensified sexual interests result in increased sexual risk-taking among adolescents. Adolescent sexual desires are influenced by increased levels of sex hormones during this time [1]. During this time, an adolescent’s need for lovemaking and intimacy with the opposite sex increases. They tend to explore different ways of lovemaking and intimacy which may put them at risk of various sexual health problems [2]. Evidence indicates that the increased prevalence of premarital sex and poor awareness of sexual and reproductive health (SRH) related matters have led to a higher risk of STI, unwanted pregnancies, abortions, and sexual violence and coercion among adolescents in the region (Bott, 2003). The findings of the National Youth Health Survey [3] implied that 14.7% of the total youth population in Sri Lanka had engaged in sexual intercourse during the previous year, out of which the majority were non-schooling youth. The survey also disclosed poor awareness among adolescents, where only 45-55% of the adolescents knew about sexually transmitted diseases (STI), contributing factors, and prevention.

A qualitative study conducted among Sri Lankan adolescents aged 17–19 years suggested that many adolescent girls were psychologically distressed due to SRH issues, the commonest being menstruation and masturbation. The same study also discovered the lack of knowledge on available sexual health services among Sri Lankan adolescents. The research pointed out the lack of access to SRH knowledge as a primary reason for SRH matters among adolescents in Sri Lanka [4].

Evidence across the world suggests that early SRH education is associated with reduced sexual risk behavior [5]. However, similar to many other countries in the Asia-pacific region, social taboos, cultural beliefs, and stigmas have become major obstacles in providing SRH education to Sri Lankan young generation [6]. As a result, SRH education of the Sri Lankan adolescents and youth are currently at a substandard level. Several attempts have been made at the national and district level during the past few decades to improve SRH knowledge among Sri Lankan adolescents. The introduction of SRH modules into the school curriculum, teacher training workshops on Adolescent sexual and reproductive health (ASRH), adolescent life skills programs, and sexual awareness programs for adolescents were some of the projects carried out at the national and district level to address the issue.

Although components of SRH have been a part of the school curriculum for nearly three decades, very little progress has been observed with the SRH knowledge, attitudes, or behaviors among the youth in Sri Lanka. The evidence suggests that only 58% of the students are satisfied with current school-based SRH education [7].

The same study found that teachers themselves preferred the subject to be taught by an external person, probably due to their lack of self-confidence in delivering the content. Lack of intense and continuous training of teachers, capacity building and underuse of interactive material, lack of support from the principals and parents have been identified as major drawbacks for a successful program. Although sexual awareness programs for adolescents and life-skills programs are being carried out by the Medical Officer of Health (MOH) level, on an ad-hoc basis, consistent delivery of such programs is not to be seen [8].

Research carried out among a sample of estate sector youth in Sri Lanka aged 18–24 years indicated that many youths depended on unreliable sources for sexual health information. Peers were the primary source of sexual health information for 55% of the participants while, they considered school teachers as the least reliable source of information [9]. Furthermore, National Youth Health Survey [3], carried out by the Family Health Bureau indicated that the knowledge on sexual and reproductive health among adolescents in Sri Lanka is not up to the expected standards. More than 54.4% of the females did not know that a pregnancy can happen during the first sexual intercourse, while only 53.3% knew that missing a period can be a sign of pregnancy. Adolescents’ knowledge of the prevention of STIs was substandard, where only 48.8% knew that consistent use of condoms is a method to prevent HIV, and only 54.8% (95% CI, 53.3–56.3) of youth correctly understood that there is a risk of transmitting STI even after a single sexual exposure.

Although several programs have been planned and implemented at the school and community levels to improve sexual and reproductive health knowledge among Sri Lankan adolescents, these programs are being implemented amidst lots of resistance from society. Social taboos and stigma towards providing sexual health information to adolescents are known challenges for successfully implementing these programs in Sri Lanka. Besides, the importance of improving parent-adolescent sexual health communication to promote healthy sexual behaviors among adolescents is a current, trending topic across the world [10–12]. Studies across the world have shown that parents can be used as a successful source of sexual health knowledge to adolescents. Literature indicate that effective parent-adolescent sexual communication can prevent unhealthy SRH practices among adolescents and promote their health [13, 14]. A systematic review that analyzed thirty-eight peer-reviewed studies on parent-adolescent sexual communication and its impact on adolescent sexual behavior showed a consistent relationship between parent-adolescent sexual communication and adolescents’ sexual attitudes and safe sex efficacy [15].

Evidence across the world suggests that poor awareness, negative attitudes, and perceived self-efficacy of the parent are barriers to parent-adolescent communication of SRH-related matters. A cross-sectional study among 394 preparatory students in central Ethiopia indicated that lack of parental knowledge, socio-cultural norms, and concern that discussions would encourage adolescent sexual behaviors, were the reasons for poor parent-adolescent sexual communication [16]. A qualitative study carried out in Sri Lanka in 2008 suggested that Sri Lankan parents are keen on providing SRH information to their adolescents. The same study found that social taboos and embarrassment are the main barriers that prevented Sri Lankan parents communicating with their adolescents about sexual health matters [17]. The Global School-Based Student Health Survey [18] carried out among Sri Lankan adolescents highlighted the parents’ inability to understand the different needs of an adolescent child. The study also pointed out the lack of parental involvement in adolescent SRH matters. Another qualitative study carried out among a sample of 17-19-year-old adolescents in Beruwala-Sri Lanka indicated that adolescents expected their parents to be more aware of adolescent SRH issues. The study also revealed that the negative attitudes of the parent were a barrier to reach the available ASRH services [19].

The available literature indicates that Sri Lankan parents refrain from discussing sexual health topics with their children for various reasons. Not being comfortable with the topic, lack of knowledge and skills to communicate this topic effectively with children were some of the reasons identified. The evidence suggest that it is important to intervene early before the adolescents become sexually active to prevent sexual risk behaviors in them. Understanding parent’s perception and concerns over communicating sexual health matters with their adolescents is essential when findings the best ways to improve SRH communication to promote healthy sexual behaviors.

The present study was conducted with the objective of exploring mother’s perceptions and concerns over sharing sexual and reproductive health information with adolescent daughters. The main aim of the study was to provide adequate information to develop an effective intervention to improve mother’s capacity to communicate SRH matters with their adolescent daughters. The study only aimed at mothers and daughters since previous literature indicate gender disparity in parent-adolescent sexual communication [20].

## Methodology

Focus group discussions (FGD) were conducted among mothers of adolescent girls aged 14–19 years in six selected MOH areas in Kalutara district from November 2019 to February 2020. Biological mothers of unmarried adolescent girls were included in to the sample. Mothers with communication difficulties, mothers who were having acute psychological disturbances that needed treatment at the time of the study and those with physical impairment and have difficulty attending the discussion were excluded from the study. Each focus group discussion in the study was limited to 10–12 participants. Six FGDs were conducted until no new data was generated and saturation was attained as suggested in literature [21].

Out of the 13 Medical officer of Health (MOH) areas in Kalutara district, six MOH areas were purposively selected by the PI considering the geographic variation in the district. In each of the selected MOH area, one public health midwife (PHM) area was randomly selected to conduct the FGDs. From each PHM area 10–12 mothers were purposively selected to include in the FGD with the help of area PHM.

Guiding rules and the FGD guide was developed after extensive literature search, with the guidance of the experts in adolescent and youth health and qualitative research field. The FGD guide consisted of ten semi-structured questions developed according to the objectives of the discussion. The guide was initially developed in English by a group of experts in qualitative research and adolescent and youth health and translated to Sinhala and back translated to English by two translators, proficient in Sinhala and English.

FGD were conducted at a venue within the PHM area that was accessible to all participants. Informed, written consent was obtained from all the participants prior to the discussion, after providing all necessary information. The Discussions were facilitated by the PI and another medical officer who had prior experience in carrying out focus group discussions as moderator and recorder. Informed verbal consent was obtained from all the participants prior to the discussion. Permission was obtained from the participants for an audio recording of the discussions. They were explained about anonymity and confidentiality assured about the privacy and confidentiality of the information given. Information about the participants’ age, religion, educational status and employment status was obtained using a separate questionnaire.

All FGDs were conducted in Sinhala language. The discussions were moderated by the PI and audio recorded. Notes were taken down manually by the assistant medical officer during the discussions. The FGD guide was used to conduct the discussion. At the end of every discussion, key points were identified, summarized, and presented to the group for further clarification. The duration of each FGD was 60–90 min. During the discussion the mothers were allowed to have interactions between them to make a comfortable and friendly environment for the discussion. The PI encouraged the free flow of thoughts among participants while keeping the discussion on track and remained neutral during the discussion to make sure that everyone feels comfortable in expressing their opinion. Active participation of all participants was ensured. Notes were taken down amidst the dialogues and after the episode with each participant to get rich qualitative data.

At the end of each FGD, the PI debriefed the discussion to ensure that none of the relevant data are missed. The moderator and the recorder went through the audio recordings and the written notes and the participant’s responses were summarized to assure saturation. The data was transcribed as soon as the FGD was completed so that nuances of the dialogues were not lost. Transcription was translated in to English by two medical officers who have proficiency in both Sinhala and English languages and checked by an external supervisor for any discrepancies. Data management and analysis mainly followed an inductive approach to thematic analysis principles [22]. Data coding was manually done by the PI and a Consultant Community physician, who had past experiences in the field of qualitative research to enhance the accuracy of the result. From the codes, categories were identified. Common themes, patterns, and relationships within responses were identified in relation to the categories developed from the codes. The analysis results were presented in narrative form using quotes in respondents’ own words that highlighted the theory. For identifying a participant, an alpha numeric number was used; E.g. B1-Mother B in FGD 1.

Credibility or the accuracy of the data gathered was ensured by prolonged engagement, persistent.

observations, and debriefing and member checks while Having clear inclusion and exclusion criteria allowed to transfer the study findings to a similar group of mothers ensuring the transferability of data. To ensure the consistency of the study findings, discussions were guided by a FGD guide that was developed with expert opinion. During the data analysis, coding was done by two data collectors separately and examined by an external expert. Sources of disagreement was identified and corrected after discussion to warrant the reliability of the results. All FGDs were audiotaped, and the notes were taken down by the recorder and summarized at the end of each discussion to confirm the data generated. Participant’s facial expressions and other non-verbal observations too were taken into consideration to ensure that emotional responses were not missed. To ensure the degree of neutrality in study findings, the moderator adhered to the FGD guide throughout the discussion. To minimize the moderator bias, the moderator had special training on conducting focus group discussions and avoided from expressing her opinion or concerns during the discussion.

## Results

### Socio-Demographic characteristics of the study participants

A total of 64 participants were included in the study. The mean age of the participants was 43.5 years. Majority (81.2%) were above 40 years. All participants were Sinhalese and out of them 82.8%, n = 53 were Buddhists, while 17.2% (n = 11) was Catholic or Christian. Out of the mothers, 34.1% (n = 40) were educated above O/L’s, while 32.8% (n = 21) were employed.

Focus group discussions conducted with the mothers of adolescent girls identified eight major themes. Among them were sexual and reproductive health issues faced by an adolescent girl, factors contributing to adolescent girls’ SRH issues, sources of SRH information to teenage girls, importance of providing SRH information to adolescent girls, mothers’ role in supporting SRH problems among teenage girls, current level of mother-daughter communication of SRH issues, challenges faced by mothers when communicating SRH problems, mothers’ suggestions to overcome the challenges.

### Themes and categories derived from focus group discussions

Focus group discussions conducted with the mothers of adolescent girls identified eight major themes. Among them were sexual and reproductive health issues faced by an adolescent girl, factors contributing to adolescent girls’ SRH issues, sources of SRH information to teenage girls, importance of providing SRH information to adolescent girls, mothers’ role in supporting SRH problems among teenage girls, current level of 221 mother-daughter communication of SRH issues, challenges faced by mothers when communicating SRH problems, mothers’ suggestions to overcome the challenges.

### Theme 1- sexual and reproductive health issues are common to adolescent girls

Mothers identified puberty related issues, issues related to romantic relationships, and exposing to sexual violence as main sexual and reproductive health concerns during adolescence. They understood that sudden physiological changes during this time can be distressful to an adolescent girl. They also understood how important the body image can be to an adolescent girl. Most of the mothers talked about the difficulties a girl could be facing due to puberty. Among these, menstruation played a major role.

My younger daughter attained puberty at the age of 12. Then she didn’t get her period for some time. When she started bleeding, she was worried about it. She was scared of blood that was coming out of her body. I told her that this is normal to any girl of her age, and then with time, she learned to accept the event

My daughter complains of pain in the upper and lower limbs, headache, and stomachache during this time. She even ignores watching TV and falls asleep the whole day through. She loses her interest in food and hates to go out of the house during this time

During this phase they undergo bodily changes like breast enlargement & increased growth of body hair that might be distressful to a girl

Many mothers did not want their daughters to be involved in romantic relationships, since they feared about the chance of intimacy. They were more concerned about the chastity of their daughters. This is largely explained by the cultural beliefs in the study setting, where virginity is given importance at the time of marriage.

We are scared that our girls might get involved in love affairs.

Love affairs can have harmful impact on an adolescent girl if her parents do not properly monitor them,

Girls will have difficulty getting into marriage if they get themselves into sexual relationships during this time

Mothers were careful to use the word ‘sexual abuse’ or ‘forced sex’ to describe sexual relationships that happen during adolescence. This could be partly attributed to the fact that that mothers were reluctant to accept that their adolescent children are willingly engaging in sexual activity. The finding can be also explained by the fact that mothers in the present study setting regarded their teenagers as small children and did not want to admit that they get sexual desires.

Pregnancies can happen during this time, mostly by forced sex,

The young girls of this age are more at risk of getting sexually abused,

If a female child gets coerced into sex once, she gets marginalized from the society and might face with recurrent abuse. This may even push her to prostitution

Mothers had a fairly good knowledge about from whom their children can get sexually coerced and the consequences of subjecting to sexual violence. They even had knowledge about the new.

ways a child can get sexually coerced such as through internet and social media.

Now there are other ways from which a girl can be put into trouble such as by posting pictures on social media without permission

### Theme 2- reasons sexual and reproductive health problems among adolescent girls

Adolescent girls’ reluctance to share SRH related matters with a trustworthy adult was identified by the mothers as a contributing factor for SRH problems in them. Some mothers worried that girls were not opening up to their parents but to their peers.

They tend to share their thoughts and feelings with friends but do not want to share those with me. If they tell their problems to us, we can help them out,

Mothers believed that lack of love and protection from the family can pushed a teenage girl into trouble.

Parents should give adequate love and care to children. Otherwise, they would go in search of love and affection from others,

Most of the time, when the mother doesn’t act as a friend to the adolescent daughter, there is a high chance that children face such issues

Mothers knew that sudden changes in hormone levels can cause increased sexual desire during this age which might put them in trouble.

*When body appearance changes with the maturation of sexual organs, a young girl might feel as if they are adults. This will probably make them try out adult behaviors, perhaps sexual behaviors*

The participants pointed out that poor SRH knowledge is one major reason for SRH issues during adolescents.

I feel my daughter is not very knowledgeable about these things. Sometimes I fear that she may fall into these risks,

If the girls know the sexual risks around them, they will try to protect themselves

They blamed school teachers for not providing their children with proper sexuality education.

Mothers also believed that children are getting exposed to sexual material that is publicized via television,

Sexual content is very much common in television and you tube nowadays. They will get the desire to practice sex after watching these

### Theme 3- sources of sexual and reproductive health information to adolescent girls

Many mothers believed that parents are a good source of sexual health information to adolescent girls.

I think parents are the best persons to deliver SRH information to young girls. Parents know what to tell their children and what not to. Also, if the parents talk to their children about these topics, children will also open up themselves to the parents

However, some mothers said school teachers are a better source of sexual health information as they know how to deliver these messages to children in the most appropriate way.

Teachers know how to teach these to the children

Some mothers would like health care workers to talk to their children regarding these matters

Schools should invite a doctor to teach this chapter so that the children will learn things better because doctors know what to tell a teen girl and how to tell.

### Theme 4- sexuality education in schools is not satisfactory

Many mothers were not satisfied with the sexuality education in schools. They complained about the fact that many teachers avoid teaching SRH chapter in schools.

Sexual and reproductive health education is there in the textbook. However, most of the teachers do not teach but give questions and ask the children to find out answers on their own after reading the book.

### Theme 5- importance of providing sexual and reproductive health information to adolescent girls

Many mothers in the present study believed it’s important to provide sexual health information to their offspring.

Giving sexual health information at an early age will minimize the curiosity of adolescents to go after sex related content

If provided with information, she will be able protect herself from any danger that might face during this time. She will also develop the capacity to solve issues she might face with

However, almost all mothers in our study group preferred “abstinence-only” sexuality education over “abstinence-plus” or “comprehensive” sexuality education. Instead, they wanted to make their girls aware of the consequences of pre-marital sex and importance of keeping body boundaries until marriage.

It is important to provide them information about the consequences of engaging in sex during this time,

If the children were made aware of these consequences they will not engage in any unwanted relationships before marriage

Nevertheless, there were some mothers who thought sexual education is not an essential requirement for the child. They feared that providing sexual health information may aggravate child’s curiosity to explore more, putting her at more risk of sex during adolescence.

I feel that giving knowledge at an early age, may arouse the curiosity of the child to explore these things more. I feel it’s good to let the child learn these things little by little naturally with time, rather than feeding her a big chunk of information at once

### Theme 6- mother has a role to play

All mothers in our study group accepted that mother should keep a healthy relationship with their daughters.

We should always be a friend to our daughters, so that they will come up with their issues to the mother

A daughter is always close to her mother. If the mother keeps the habit of talking to her daughter about every day information, she will not hesitate to open up with any of her confidential matters to the mother.

### Theme 7- communicating SRH matters with daughters

It was seen that majority of the mothers in our study group realized the importance of making their children aware of sexual health matters and were trying to communicate about sexual health matters.

*Sometimes it’s not easy. But I try hard to keep her informed about the sexual risks she may have to face and how to protect herself from them*

For many mothers this was a difficult topic to communicate with their children.

There are things that we cannot tell in depth

Issues related to menstruation, preventing sexual violence, chastity, and sexual abstinence were the commonly discussed topics between the mother and daughter.

I have discussed with my daughter about problems related to menses. I ask about the duration of the bleeding and check whether it is regular

I have told my daughter to inform me if a stranger touches her body and not to open the door when she is alone at home

I often advice my daughter not to let anyone see or touch her private parts

Mothers had warned their adolescent girls about having romantic relationships and had advised their daughters to avoid sex within a relationship. They used indirect words such as ‘*unwanted things,’ ‘close contacts’* to depict sex within a relationship, which showed the mothers’ embarrassment over using direct language.

*I have told my daughter that if she starts a romantic relationship with a man and if an unwanted thing happens, she will never get a chance to marry another man. I have told her that she has to protect herself until marriage*

My girl has a boyfriend. I have told her it’s okay to have a boyfriend but I advise her not to have intimacy. I have made her aware of the adverse things that can happen if she gets into an intimate relationship

Although mothers wanted to communicate sexual health matters with their adolescent daughters, they were reluctant to communicate some sexual matters.

I have not told my daughter about the sexual act or how a conception occurs. It’s embarrassing

At times they provided dishonest answers to questions raised by their children.

I have told her that babies are born from a cut made on the abdomen

### Discussing about contraceptive methods with your daughter is not a good decision

Many mothers in our study sample did not agree with discussing about contraceptive methods with their children.

I don’t think it’s a good decision to make them aware of the contraceptive methods

### Theme 8- common techniques used by mothers to communicate sexual and reproductive health issues with adolescents

According to the present study, many mothers who had been discussing SRH matters with children, have been doing so, while watching or listening to incidences of sex-related matters.

I find opportunity to discuss these matters with my daughter when we are watching television. When they report about sexual abuse incidences in news I teach about these things

When I see sexual violence cases reported in news-papers I ask her to read too. Then she comes up with questions

### Theme 9- mothers’ concerns about providing SRH information to adolescents

#### Adolescents need to be taught about the negative consequences of having premarital sex

Majority of the mothers in the present study believed that provision of sexual health information to adolescents should be done within limits. They were more concerned about the chastity of their female offspring.

The girls should be made aware of the negative effects of having sexual relationships, during this time

They wanted to make their girls aware of the consequences of pre-marital sex and importance of keeping body boundaries until marriage.

It is important to tell the children what bad sex before marriage can do to a young girl, during this time

Mothers believed that too much information may cause unnecessary fear in children.

In my daughter’s school they had shown some videos and discussed some cases of sexual abuse with our children. These cases were about rape and crimes that happened to children of that age. My girl was frightened after seeing these videos and hearing the stories thinking that these might happen to her too.

### Sexuality information should be provided to children in step-by-step manner, age appropriately

Mothers suggested of a step-by-step approach to provide SRH information to teenagers.

*If the children were provided this information in a step-by-step manner, this would not have happened, but when the information is given all at once the children get feared or dismayed*,

They were also concerned about providing this information appropriate to the age of the girl.

I think information about menses should be given at an early age around 10 or 11. Information about sexual violence, consequences of premarital sex should be given at an age earlier than 15 years, probably at 13–14 years. Information about family planning methods must be given at a later age, probably at the age of 18–20

### Family Planning information should not be shared with children

Mothers believed that providing contraceptive information to children should be done with cautious since it may encourage children to engage in sexual behaviors at an early age.

I have never discussed about family planning methods with my daughter. I think making them aware of contraception might encourage them to try these methods and to engage in these activities without fear

### Provision of sexual and reproductive health information should be individualized to the needs of the child

Mothers in our study group also believed that sexual information should be individualized to the needs of their children.

I think before we give knowledge and information to our children, we should know clearly what our girls are up to. There are some children who would want to protect themselves from these risks. But there might also be some others who try to experience these,

### Talking about sexual health matters would encourage children to explore sex

Some mothers avoided talking to their children about sex thinking that communicating sex may increase child’s curiosity to explore about sex more.

I feel that talking about these things would aggravate a child’s curiosity to explore these things more

### Talking about sexual matters may lose child’s respect towards the parents

Some of the parents in our study assumed that children may lose respect towards their parents if they talk more informal topics like “sex” with their children.

I’m afraid, she might think I’m okay with these things if I talk to her about these topics

### Escaping from the responsibility to educate their children on SRH

Some mothers wished their children would get knowledge about sexual health from other sources.

I don’t know how to discuss these things with children. Rather, I wish they learn from their peers and other sources

### Theme 10- barriers to communicate SRH topics with daughters

#### Difficulty responding to questions

Mothers feared if their children would come up with questions when they communicate sexual health matters

Children sometimes ask how women get pregnant. Then it’s actually difficult for us to answer. Sometimes we lie to them.

#### Difficulty using direct language

They had difficulty in using direct language when communicating sexual health matters with children.

I don’t use the exact words when talking about these things. It’s embarrassing

#### Inadequate skills and knowledge to communicate sex with children

Many mother participants were uncertain whether they are practicing the correct way of communicating sexual matters with children.

I’m concerned whether my way of telling these things is the appropriate way to communicate these things with a young child. I’d rather keep my mouth shut

Some mothers doubt whether they have adequate knowledge and skills to communicate these matters with children.

I want to teach my girl about these things, yet I’m not sure whether I have enough knowledge

I don’t discuss these things with my daughter because I don’t know how to do it

I fear about the questions she might raise, if I start a discussion with her on this subject

### Shame and embarrassment around the topic

Embarrassment was one other factor that prevented mothers from discussing sexual matters with their offspring.

Yes, we should answer their queries. Still, at times I’m unable to do that. Sometimes I know the answer but, I’m shy to tell these things to my daughter. However, it’s still good if we can give them accurate information

### Adolescent girls’ reluctance to open up to their parents

Mothers also talked about the fact that their adolescent daughters are reluctant to open up to their parents regarding this topic

These girls sometimes try to hide their feelings. Some girls tell only a little and the rest they refrain from telling to parents.

### Theme 11- mothers’ suggestions to overcome the challenges of mother-daughter SRH communication

#### Mothers need to be educated about delivering SRH information to children

Mothers wanted to know more about SRH issues faced by an adolescent girl. They also wished they knew the correct method to deliver SRH messages to children at the appropriate age.

*I need to improve my knowledge on these things more*

I think I have both knowledge and skills to discuss these things with my daughter. But, I have not talked to my daughter about these things because I don’t know when to discuss these things with her

mothers in the present study agreed that they need to be equipped with the knowledge and necessary skills to discuss these matters with their daughters effectively.

*“If mothers can be trained on how to tell and what to tell according to the child’s age that would be very useful”* mothers found it easier to discuss sexual matters with the children when they had prior sexuality education in school.

## Discussion

In contrary to similar studies conducted in South Asia, which indicated socio-cultural norms were a main barrier to provide sexuality education to children [23], many mothers in the present study believed it’s important to provide sexual health information to their offspring. This can be partly explained by the high literacy rate in the context of the study that made the adults to realize the importance of sexuality education to children. At the same time, mothers had their own fears about providing sexual health information to adolescent girls believing that it might aggravate child’s curiosity to explore more, putting her at more risk of sex during adolescence. The finding was not strange to the present study setting, since similar findings have previously been reported by many other research that were carried out in the context of the South Asia, where parents were concerned about experimentation and possible early sexual exposure due to sexual education [23, 24].

Majority of the mothers in the present study believed that provision of sexual health information to adolescents should be done within limits. They preferred abstinence-only sexuality education over abstinence-plus or comprehensive sexuality education. The finding was similar to many other studies conducted in South Asia and similar study settings, that promoted abstinence only education. Focus group discussions (FGD) carried out among parents of adolescents aged 15–18 years in Fiji identified that parents limited their discussions to advice children about not engaging in sex (Ram et al., 2020). A systematic review that looked over 29 studies across the world, indicated that parents from countries like Bangladesh and Malaysia preferred sexual education to be in line with religious teachings, values and cultural context. Nonetheless, evidence from western cultures have indicated failure of abstinence only education [25], in preventing teenage pregnancy.

Mothers in the present study found it easier to discuss sexual matters with children when they had prior sexuality education in school. Available evidence indicates that when the children participate in school based sexual health education programs, sexual communication between the parent and adolescent increases [26].

Issues related to menstruation, preventing sexual violence, chastity, and sexual abstinence were the commonly discussed topics between the mother and daughter. Similar findings had been reported in a previous study carried 225 out among 1174 female students in Bangladesh where it was found that > 80% of the girls discussed menstrual issues with their mothers [27].

Mothers in the present study setting regarded their teenagers as small children and did not want to admit that they get sexual desires. Above finding conforms with the results of a cross-sectional study that was conducted among 186 mothers and father in US, where many mothers considered their adolescent children as ‘too young’ to engage in sexual activities [28]. The particular study identified this as a major barrier for sexual discussion between mother and daughter.

According to the present study, many mothers who had been discussing SRH matters with children, have been doing so, while watching or listening to incidences of sex-related matters. Similar findings have been reported in a study carried out in Bangladesh in the year 2020, in which it was found that mothers who had good media use such as newspapers and television were having a high level of SRH communication with their daughters [27]. The finding reflects the importance of IEC material to explain SRH topics to children, which mothers are not comfortable with.

Similar to the present study findings, FGDs conducted among 71 parents in Kurunegala indicated that parents were reluctant to discuss SRH matters with children mainly due to shyness, embarrassment, and lack of knowledge [17]. Moreover, a study conducted among 186 nationally representative sample of US mothers and fathers reported that lack of self-efficacy and perceived value in sex communication are factors determining mother-daughter sex communication [28].

### Implications of the findings

The parents’ interest in developing a sexually healthy adolescent can be well used to plan public health programs to improve SRH awareness among children and adolescents. The unconditional love towards the child, trustworthiness, and the ability to offer developmentally appropriate, individualized information consistently makes the parent a reliable and tailored source of sexual health information to children. Moreover, the ability to adapt the messages to suit the religious, cultural, and family values would make this approach more acceptable in the society, where there is much resistance against providing sexuality education to children.

Even though many mothers had recognized the importance of mother-daughter sexuality communication and had taken the first steps already, there were exceptions in the society who had not realized the need to provide sexual health information to adolescents. The findings indicated the need to carry out public health programs targeted at parents to improve their knowledge and attitudes towards the sexuality education of children. Furthermore, out of the mothers who had been doing something to address the issue, the majority were ignorant about what, when and how to communicate sexual matters with children. The finding indicates the need to develop parents’ capacity in sharing SRH information with children.

The present study identified the main areas that need to be focused on during future public health interventions targeting parents and adolescents to promote ASRH. Among them were; the need to make a positive change in parent’s attitudes towards sexuality education of adolescents, improving parent’s knowledge on SRH needs of an adolescent child, need to develop communication skills of the parent, need to develop the adolescents’ capacity to identify correct sources of sexual health information and to seek help when faced with SRH issues.

School-based and community–based interventions have to be targeted to cover the above objectives. Advocacy programs targeting education staff, including school principals to get the active involvement of the parents during school sexuality education sessions is also a requirement.

### Conclusions and recommendations

Policymakers need to consider parents as a primary source of sexual health information for adolescent girls. Implementation of parent awareness and skill-building sessions in parallel to the school-based sexual health education programs is recommended. The use of print, local, and mass media messages to create awareness among mothers on adolescent SRH matters and mothers’ role in supporting adolescent SRH issues is suggested.

Mothers used material such as newspaper articles, movies, TV shows, and text-books to explain SRH matters to children. The development of video clips and other IEC material, which can be used by the parents and teachers when giving SRH education to children, is recommended.

Mothers wanted to give abstinence-only sex education to their adolescent children, where they thoroughly refused to provide safe sex information to adolescents. Hence, future interventions should focus on improving mothers’ attitudes towards the sexuality education of adolescent children. Furthermore, mothers perceived school-based sexuality education is not up to the level of expectation, and the reluctance of the school staff to provide SRH information to children. Interventions targeted at school principals and other relevant staff on improving their knowledge and attitudes towards sexuality education, skill building sessions for the teachers are recommended.

### Future research

The present research only targeted at exploring mother’s concerns over sharing sexual health information with their daughters. Future research should be conducted to examine the father’s concerns over sharing sexual health information with adolescent children including the sons.

## Electronic supplementary material

Below is the link to the electronic supplementary material.

Supplementary Material 1: Focus Group Discussion Guide

## Data Availability

The data generated during this study are included here as a supplementary file. The audio recordings of the focus group discussions are available from the corresponding author on reasonable request.
