# Supplementary material for: Mother’s perceptions and concerns over sharing sexual and reproductive health information with their adolescent daughters- A qualitative study among mothers of adolescent girls aged 14–19 years in the developing world, Sri Lanka
Source: BMC Womens Health. 2023 May 3;23:223. doi: 10.1186/s12905-023-02369-1 (PMC10157993; doi:10.1186/s12905-023-02369-1)
Supplement: Supplementary file 1 — Supplementary Material 1: Focus Group Discussion Guide [file 12905_2023_2369_MOESM1_ESM.docx]

**Annexure 2**

**The focus group discussion guide**

- 1. What do you think are the common sexual health issues faced by female adolescents?
  2. What do you think are the underlying factors leading to these issues?
  3. What do you think about the need of providing adolescent girls with sexual and reproductive health information?
  4. What do you think are the common sources of sexual and reproductive health information for adolescents?
  5. What do you think is the parent’s role in preventing these sexual health issues?
  6. Have you ever talked to your daughters regarding sexual health topics?
  7. How have you carried out these discussions?
  8. If so, what topics have you discussed with your adolescent?
  9. What challenges have you faced when discussing sexual and reproductive issues with your adolescent girl?
  10. What would make it easier for the mothers to discuss SRH issues with their children?

**FGD Rules**

1. Arrive at least one hour earlier to set up the room
2. Test your recording equipment, be ready with pens and note-pads
3. Welcome the participants
4. Introduce yourself, your assistants. Explain the purpose of the focus group discussion.
5. Explain the participants that they have been invited to share their opinion on communicating sexual and reproductive health topics with their girls and that you will be guiding them by asking specific questions.
6. Explain the privacy and confidentiality of the information.
7. After explaining the above, ask for their consent in participating in the discussion.
8. Explain the ground rules of the discussion
9. Participation in the discussion is voluntary.
10. You can abstain from discussing certain topics if you don’t wish to
11. You can come up with any of your opinions. All responses are valid. There are no right or wrong answers.
12. Respect others opinion during the discussion, even if you don’t agree.
13. Try to stay on the topic; We may answer your questions at the end of the discussion
14. Speak as openly as you are comfortable.
15. We don’t expect you to reveal very detailed information about your personal life or health
16. Help to protect the privacy of others by not discussing the information outside the group.

When facilitating the discussion,

1. Ask open-ended questions. Make the environment non-threatening that will enable everyone to come up with their opinion comfortably.
2. Encourage all the participants to participate in the discussion.
3. Pay attention to non-verbal cues.
4. Make sure that you don’t share your own opinion or favor one participant over the other.
5. Redirect participants when the discussion strays far off from the topic.
6. Check with the participants that you understood what they were saying

Closing the session

1. End the discussion by summarizing the main points
2. Thank the group for participating. Explain to them how the discussion results will be used.
3. Collect all the notes and recordings
